# Supplementary material for: Spatial stratified heterogeneity analysis of field scale permafrost in Northeast China based on optimal parameters-based geographical detector
Source: PLoS One. 2024 Feb 16;19(2):e0297029. doi: 10.1371/journal.pone.0297029 (PMC10871524; doi:10.1371/journal.pone.0297029)
Supplement: S1 Table — (DOCX) [file pone.0297029.s001.docx]

**S1 Table. q values of variables by factor detector in each highway.**

| **Highway** | **q values of variables** | | | | | | | | | |
| --- | --- | --- | --- | --- | --- | --- | --- | --- | --- | --- |
|  | SFN | γ | DEM | TD | ASCD | RN | ASPECT | SLOPE | SOC | SCF |
| **WX** | 0.1042 | 0.1662 | 0.0819 | 0.0698 | 0.0994 | 0.0746 | 0.0263 | 0.0851 | 0.0363 | 0.0576 |
| **GM** | 0.0586 | 0.1150 | 0.0534 | 0.0198 | 0.0380 | 0.0263 | 0.0576 | 0.0159 | 0.0314 | 0.0518 |
| **JC** | 0.1644 | 0.0995 | 0.1213 | 0.1168 | 0.1164 | 0.0905 | 0.0357 | 0.0945 | 0.0326 | 0.1257 |
| **KG** | 0.1402 | 0.1060 | 0.0862 | 0.2643 | 0.1202 | 0.1527 | 0.1592 | 0.1562 | 0.0922 | 0.1381 |
| **SL** | 0.0185 | 0.1231 | 0.0474 | 0.0465 | 0.0290 | 0.0147 | 0.0174 | 0.0146 | 0.0179 | 0.0158 |
| **YC** | 0.0889 | 0.1326 | 0.3478 | 0.0683 | 0.4158 | 0.0467 | 0.0853 | 0.0405 | 0.0617 | 0.1386 |
| **TG** | 0.0833 | 0.1104 | 0.0317 | 0.0163 | 0.0145 | 0.0255 | 0.0139 | 0.0248 | 0.0136 | 0.0275 |
| **Highway** | **q values of variables** | | | | | | | | | |
|  | SBD | CHILI | mTPI | NDVImax | NDVImean | DEV | TPI | VEG | LF |  |
| **WX** | 0.0085 | 0.0596 | 0.0329 | 0.0459 | 0.0448 | 0.0373 | 0.0258 | 0.0304 | 0.0653 |  |
| **GM** | 0.0325 | 0.0123 | 0.0363 | 0.0090 | 0.0096 | 0.0632 | 0.0327 | 0.0211 | 0.0288 |  |
| **JC** | 0.0949 | 0.0691 | 0.1099 | 0.0396 | 0.0447 | 0.0525 | 0.0696 | 0.0026 | 0.0939 |  |
| **KG** | 0.1221 | 0.2033 | 0.1030 | 0.0523 | 0.0245 | 0.1320 | 0.1016 | 0.0431 | 0.1038 |  |
| **SL** | 0.0251 | 0.0187 | 0.0331 | 0.0087 | 0.0129 | 0.0056 | 0.0207 | 0.0661 | 0.0282 |  |
| **YC** | 0.0328 | 0.0185 | 0.0648 | 0.0216 | 0.0320 | 0.0188 | 0.0359 | 0.2322 | 0.0088 |  |
| **TG** | 0.0380 | 0.0115 | 0.0065 | 0.0347 | 0.0461 | 0.0150 | 0.0095 | 0.0551 | 0.0254 |  |
