# Supplementary material for: Spatial stratified heterogeneity analysis of field scale permafrost in Northeast China based on optimal parameters-based geographical detector
Source: PLoS One. 2024 Feb 16;19(2):e0297029. doi: 10.1371/journal.pone.0297029 (PMC10871524; doi:10.1371/journal.pone.0297029)
Supplement: Attachment — Submitted filename: Response to Reviewers.docx [file pone.0297029.s002.docx]

Response to Reviewers

**Summary**

Dear reviewers,

I wish to express my gratitude for your meticulous review and invaluable suggestions. We deeply appreciate the opportunity to learn from your extensive knowledge and experience. Your expertise and detailed evaluation have significantly contributed to the enhancement of our research. We have carefully considered and implemented each of your suggestions in the revised manuscript.

The highlighted portions indicate the changes made in the manuscript. The overall structure of the article has been adjusted, particularly in the discussion section where the content order has been rearranged, and additional material has been included. Additionally, to enhance the manuscript's conformity, some textual expressions have been adjusted without altering the original meaning; these sections are not specifically highlighted.

The revisions based on your suggestions have been thoroughly incorporated into the revised version, which can be found in the resubmitted manuscript. Thank you once again for your time and patience. We eagerly anticipate your feedback.

Best regards,

1. **Point-by-point response to reviewer 1**

**1.1 Comments 1: Lines 53-54，I suggest the authors add relevant data to demonstrate the severity of permafrost degradation.**

**Response 1:** Thank you for pointing this out. We have incorporated the relevant data as per your request (Lines 43-45).

**1.2 Comments 2: In the last paragraph of the introduction, I suggest the authors further highlight the significance of this study.**

**Response 2:** Agree. We have added textual descriptions as per your request to emphasize the significance of this study (Lines 100-104).

**1.3 Comments 3: To present the figures more clearly, the authors should change the latitude and longitude of Figure 1 to English expression.**

**Response 3:** I apologize for the oversight. It has been rectified.

**1.4 Comments 4: In GD model, why didn't the authors add an introduction to the detection of multiple ecological factors?**

**Response 4:** You are correct; the GD model includes the detection of multiple ecological factors. In our experiments on ecological detection, the results indicated significant differences among all variables, suggesting a lack of correlation between them. This could be attributed to the large sample size, making the F-test inappropriate. The outcomes of ecological detection did not contribute to the analysis of the relationship between permafrost and environmental variables. Consequently, we did not include a detailed discussion of the detection of multiple ecological factors.

**1.5 Comments 5: Why didn't the author consider temperature and precipitation when selecting influencing factors?**

**Response 5:** Thank you. This is because existing high-resolution temperature and precipitation data are often obtained through interpolation or model integration, combined with ground data calibration, rather than being directly observed. This paper primarily focuses on the performance of permafrost at the field scale, aiming for environmental variables that have high resolution and are directly observed or measured. Therefore, temperature and precipitation were not considered. We will take your suggestion into account in future research.

**1.6 Comments 6: In order to further highlight the innovation of this article, it is better to compare the results of this study with other studies.**

**Response 6:** Thank you. We agree with your suggestion and have enumerated and compared various methods used in the NEC region and studies conducted at different scales in the introduction (Lines 61-63, Lines 66-71). This highlights the innovative aspect of our research, which seeks to reconcile scale and scope.

**1.7 Comments 7: A paragraph of limitation discussion should be added to clarify the limitation or uncertainty of current study. For example, the uncertainty of remote sensing data including NDVI data (e.g. Ma et al., 2022; Shen et al., 2022) may affect the research results.**

**Response 7:** As you suggested, we have added Section 4.4 in the discussion section to explore the limitations of this study, including the uncertainty associated with SFN, ASCD, and NDVI data, as well as the line-based spatial distribution of samples.

**1.8 Comments 8: In discussion, more mechanism explanations should be added to further explain the relationship between permafrost degradation and terrain, climate, and vegetation.**

**Response 8:** Thank you for your update. Variables closely related to terrain include TD, RN, γ, and slope. In the revised manuscript, specifically in lines 301-307 and lines 368-373, we have explored the relationship between permafrost degradation and terrain from two different perspectives. Additionally, in lines 311-320, we have provided an explanation of the vegetation response mechanisms to permafrost.

We apologize for the challenges in conducting climate analysis at such a small scale. However, we have addressed temperature and moisture influences on permafrost preservation in the interpretation of vegetation and snow-related parameters, as well as in the discussion of the permafrost distribution characteristics along various roads, such as YC (lines 426-428).

**1.9 Additional response:** In response to the suggestions from both the reviewers and the editor, we have restructured the discussion section of the manuscript to enhance its coherence and readability. We have incorporated the valuable references mentioned by the reviewers, specifically addressing the vegetation change processes in the study area. This addition is now located in the discussion section (Lines 311-313).

2. **Point-by-point response to reviewer 2**

**2.1 Comments 1: The content structure needs to be adjusted, e.g., the content of line358-366 should be adjusted to the methodology section.**

**Response 1:** Thank you for your update. To enhance the clarity of the manuscript's structure, we have relocated the Receiver Operating Characteristic (ROC) test to the methods section (Section 2.2.4), as you suggested. Additionally, we have adjusted the structure of the discussion section to improve the overall coherence and readability of the paper.

**2.2 Comments 2: Insufficient care and interpretation of the results of the risk detectors was carried out only on the magnitude of the resultant values. The risk detector focuses on detecting whether spatial patterns based on mean representations are significantly different between subregions categorized by categorical or hierarchical variables. This component needs to be strengthened.**

**Response 2:** Thank you for pointing this out. To ensure sufficient care and interpretation of the results of the risk detectors, we have added an explanation of the risk detector outcomes in Section 4.1. This includes highlighting the differences in permafrost risk values across different variable value ranges and elucidating the mechanisms causing these variations. This addition aims to underscore the distinctions between subregions categorized by categorical or hierarchical variables.

**2.3 Comments 3: Given the spatial heterogeneity, it is rather unfortunate that the authors did not address the effects of zonation, such as longitude and latitude, since there is a certain span of latitude and longitude for each highway.**

**Response 3:** Thank you for the suggestions. We have strengthened the analysis and discussion in this section. In Section 4.3 of the paper, following a reorganization, we have compared the spatial locations of different highways, their geographical environmental conditions, and the resulting spatial heterogeneity. Furthermore, we have discussed the mechanisms behind these observations.

**2.4 Comments 4: When using the geodetector method, the pursuit of a large q-value is one of the goals, but the choice of segmentation points for variables needs to be based on practical significance and not on the size of the q-value. For example, the choice of R when calculating TPI and DEV (line224-278) needs to take into account the difference in actual spatial resolution, the difference in spatial resolution between the explanatory factor variables and the results based on the highway survey report.**

**Response 4:** Thank you for pointing out. Indeed, the choice of segmentation points for variables should be driven by practical significance rather than the size of the q-value. The principle of the optimal parameters-based geographical detector (OPGD) involves comparing different segmentation approaches to identify, for each variable, the one that provides the strongest explanatory power, i.e., the optimal q-value segmentation. In this study, given the limited research experience on the segmentation of many variables, we opted to use the optimal geographic detector to determine variable intervals.

We aimed for TPI and DEV to be two variables reflecting the relative trends in soil moisture accumulation. The observed distribution patterns of permafrost in the NEC have confirmed its correlation with soil moisture. However, there is a lack of sufficient research data on how the degree of moisture accumulation affects permafrost existence and the variations in impact across different moisture value ranges. This study aim to explore the differences in the impact of moisture values in different intervals on permafrost existence. Therefore, the selection and segmentation of R for TPI and DEV are optimized with the goal of maximizing the q-value..

**2.5 Comments 5: As far as I know, the discretization method when optimizing a geodetector also includes the standard deviation (sd), why did the authors discard it. (line147-148).**

**Response 5:** Thank you, yes, you are right. Standard deviation (sd) is one of the discretization methods used in optimizing a geodetector. However, in our experiments, we found that standard deviation (sd) cannot be applied. This might be due to the large volume of data, making it challenging to ensure that the discretization method can effectively distinguish within the accuracy range. Therefore, we have chosen not to include sd as one of the discretization methods.

**2.6 Comments 6: In the results section it is recommended to give the results of the q-values (in the form of graphs or tables) for all variables for each highway, instead of the so-called highest degree of interpretation in the article. This is because the differences between the q-values are also important and need to be analyzed for this aspect of the content.**

**Response 6:** Certainly, According to your request, we have incorporated the results of the q-values for all variables for each highway into the supply data. Additionally, we have included a description of these data in the results section (Lines 261-265).

**2.7 Comments 7: I am curious as to whether the authors used a certain range of mean values for the raster data extraction when pre-processing the geodetector input data, or were the values extracted directly by geographical location? The question still connotes the issue of spatial resolution differences between the data.**

**Response 7:** In this study, a spatial scale of 30m was adopted as the primary scale for analysis. This choice is attributed to the fact that the majority of the data possesses a resolution of 30m. Additionally, during the preprocessing of geodetector input data, the values of variables were directly extracted based on geographical location. This approach was employed because we aimed to analyze the variability in the interpretive capability of environmental variables at the smallest possible scale. Consequently, the decision was made not to compute the mean values, emphasizing a focus on finer-scale differences.

**2.8 Comments 8: How to account for large differences in both time and space in the input variables in a way that does not lead to less feasible or even erroneous results.**

**Response 8:** It is indeed true that significant differences exist in both the temporal and spatial aspects of the input variables. To address differences in the time scale of variables, several considerations were taken into account in the processing of time-series data, including SFN, ASCD, NDVImean, and NDVImax.

1. SFN:** This variable, characterizing the annual surface freeze-thaw status, is derived from remote sensing data. In this study, SFN data from 2014 to 2019 were selected. This timeframe aligns well with the EGIR reporting period, and the stability and consistency of SFN across various highways during this period have been documented [1].

2. NDVImean and NDVImax:** Considering the rapid vegetation changes typically observed 2-3 years after permafrost degradation in the NEC [2], NDVImean and NDVImax were computed as the average values for the 3 years preceding the EGIR reporting time.

3. ASCD:** Unlike NDVI, which is indicative of permafrost degradation, snow affects the thermal conductivity of the ground and air, influencing permafrost degradation. Given the substantial year-to-year variability in snow days, a 10-year average was computed to obtain a relatively stable value, allowing for an exploration of the relationship between snow and permafrost.

Despite the differing temporal scales of these variables, their analysis enables a comprehensive examination of their relationships with permafrost.

Regarding differences in spatial scale, the resolution of variable data are the highest obtainable resolution. To ensure the data's authenticity, originating from actual observations or calculations based on real observations, these variables were selected based on their highest available resolution.

**2.9 Comments 9: Some expressions need to be adjusted, e.g., lines 136-138, ①, ②, ③, please delete; line 155, where a capital W is not required, same elsewhere; numbers one to nine without units should be presented as words; numbers 10 and over without units should be presented as numerals; and all numbers with units should be presented as numerals (e.g., line 174, same elsewhere).**

**Response 9:** Thank you for your suggestion. We have made the modifications according to your requirements. These suggestions are very helpful for improving this article and future writing.

**2.10 Comments 10: It is recommended that the language of the article be polished to improve readability.**

**Response 10:** Thank you for your comment. We have made every effort to correct inappropriate expressions and grammar errors to ensure that the revised manuscript meets the standards.

**2.11 Comments 11: Some minor flaws and suggestions:**

**1. Line 21. The words “global climate change” suggest directly “global warming”.**

**2. Line 30. Suggests giving the definition of H-factor in the abstract section.**

**3. Line 46-47, give references for permafrost definition.**

**4. Line 192, “selection” should be “selecting”.**

**5. Line 252-253, table3, cm3/cm3 should be cm3/cm3. * Please change to Note: , same elsewhere.**

**6. Line 337, SLOPE, no need to capitalize, same elsewhere.**

**7. References, please adjust the formatting and writing style to be consistent, e.g. Line 431, Line 61.**

**8. Figures, there are Chinese characters in Figure 1. γ is written incorrectly in Fig. 2 and Fig. 6, if it is a size, it should be Γ.**

**Response 11:** We greatly appreciate your thorough reading and assistance in improving the quality of our manuscript. We have made all the necessary revisions as per your suggestions. Thank you for your valuable input.

**2.12 Additional response:** Thank you for the detailed editing suggestions provided for our manuscript. Your professional and meticulous advice has been highly beneficial. Following your recommendations, we have made adjustments to the structure of the paper, especially in the discussion section. Simultaneously, we have removed some inefficient expressions and added in-depth discussions on the methods and mechanisms of the phenomena.

[1] Shan W, Zhang C, Guo Y, Qiu L, Xu Z, Wang Y. Spatial Distribution and Variation Characteristics of Permafrost Temperature in Northeast China. Sustainability. 2022;14(13):8178.

[2] Jin X-Y, Jin H-J, Iwahana G, Marchenko SS, Luo D-L, Li X-Y, et al. Impacts of climate-induced permafrost degradation on vegetation: A review. Advances in Climate Change Research. 2021;12(1):29-47.
